# Caregiver Burden and Psychological Distress Among Informal Caregivers for Individuals with Dementia in the Republic of Kazakhstan: A Cross-Sectional Study

**DOI:** 10.3390/healthcare13202633

**Published:** 2025-10-20

**Authors:** Yelaman Toleuov, Ken Inoue, Kamila Akkuzinova, Timur Moldagaliyev, Nursultan Seksenbayev, Ulzhan Jamedinova, Haruo Takeshita, Yasuyuki Fujita, Nargul Ospanova, Altay Dyussupov

**Affiliations:** 1 Department of Psychiatry and Narcology, Semey Medical University, Semey 071400, Kazakhstan; elamantol96@gmail.com (Y.T.); akkuzinova@gmail.com (K.A.); timur_party@inbox.ru (T.M.); nurs_7sk@inbox.ru (N.S.); nargul_ospanova@mail.ru (N.O.); 2 Research and Education Faculty, Medical Sciences Cluster, Health Service Center, Kochi University, Kochi 780-8520, Japan; 3 Center of Nuclear Medicine and Oncology, Semey 071407, Kazakhstan; u.jamedinova@gmail.com; 4 Department of Legal Medicine, Faculty of Medicine, Shimane University, Shimane 693-8501, Japan; htakeshi@med.shimane-u.ac.jp (H.T.); yfujita@med.shimane-u.ac.jp (Y.F.); 5 Board, Semey Medical University, Semey 071400, Kazakhstan; altay.dyusupov@smu.edu.kz

**Keywords:** dementia, caregiver burden, depression, anxiety, stress, Zarit Burden Interview (ZBI), Depression Anxiety Stress Scale-21 (DASS-21), Kazakhstan

## Abstract

**Background:** In the Republic of Kazakhstan, informal caregivers remain the main source of patient support. Caregivers of individuals with dementia experience an increased caregiver burden and significantly higher levels of stress. We investigated the levels of caregiver burden, depression, anxiety, and stress among informal dementia caregivers and assessed the relationships between these indicators and the caregivers’ socio-demographic characteristics. **Methods:** With the cooperation of two regional mental health centers and three polyclinics in Kazakhstan, we conducted a cross-sectional study of 174 informal caregivers of dementia patients. The caregiver burden was assessed using the Zarit Burden Interview, and psychological distress was measured with the Depression Anxiety Stress Scale-21. The caregivers’ sociodemographic data were collected and analyzed. The adjusted odds ratios and 95%CIs were calculated using logistic and ordinal regressions. **Results:** Overall, 75.9% of the caregivers reported experiencing at least a mild burden, and the levels of depression, anxiety, and stress among the caregivers were higher than expected. Spousal caregivers had higher odds of abnormal depression (*p* = 0.022) and anxiety (*p* = 0.020); in severity models, spouses more often had a severe burden (*p* = 0.042) and anxiety (*p* = 0.006). **Conclusions:** Caregiver burden and psychological distress are highly prevalent among informal dementia caregivers in Kazakhstan, particularly among spouses. It is especially important to provide support for spouse caregivers. Culturally tailored psychoeducation, support groups, and respite services are urgently needed to mitigate the mental health risks faced by dementia caregivers in Kazakhstan.

## 1. Introduction

The global population as of this writing is approx. 8232 million, and it is expected to rise to 8569 million by 2030, 9177 million by 2040, and 9664 million by 2050 [1]. According to the World Health Organization (WHO), 57 million people worldwide suffered from dementia in 2021. Dementia is currently the seventh leading cause of death worldwide and is a major contributor to disability and dependence among older adults, often leaving them in need of continuous external assistance [2]. The majority of dementia patients receive care from family members and close relatives. Several studies have indicated that caregivers of individuals with dementia experience considerably higher stress levels than those caring for non-dementia patients; they are also at a greater risk of developing depression and physical health problems [2,3,4].

Zarit et al. defined caregiver burden as “the extent to which caregivers perceived their emotional or physical health, social life, and financial status as suffering as a result of caring for their relative” [5]. Several factors are known to contribute to caregiver burden, including caring for an elderly person or another individual who is completely dependent on external help, and caring for someone with mobility or behavioral issues. These caregiving responsibilities have been shown to be closely related to higher levels of stress and caregiver burden; moreover, living and working conditions significantly impact caregivers [6].

A recent systematic review of meta-analyses including studies from virtually all regions of the world revealed that the median prevalence of caregiver burden among informal caregivers was 49.26% [7]. A study conducted in 2023 in Austria identified the most significant factors influencing the caregiver burden as poor caregiver health, behavioral issues in the care recipient, extensive caregiving hours, and a high level of patient dependence [6]. A review conducted in Singapore reported that the prevalence of caregiver burden among those caring for dementia patients ranged from 23.0% to 59.2% [8]. In Malaysia, the prevalence of caregiver burden among family caregivers of people with dementia was 69.4% [9]. Kim et al. observed that the greater the severity of the patient’s behavioral and psychological symptoms of dementia, the higher the caregiver burden experienced by family members [10]. In a study from Japan, the costs of informal care were associated mainly with the employment status of the caregivers [11]. That study also showed that (*i*) out-of-pocket payments for long-term care were associated with care-need levels, family economic status, and (*ii*) activities of daily living (ADLs) and instrumental activities of daily living (IADLs) such as bathing, toileting and cleaning were associated with the overall costs [11]. An investigation of caregivers in France, Germany, Italy, Spain, the UK, and the U.S. showed that combinations of behavioral symptoms could have a detrimental impact on health-related outcomes such as healthcare resource use, patient quality of life, and caregiver burden [12]. A study of caregivers in Brazil revealed that sleep disturbances among caregivers were correlated with higher burden levels and contributed to more vulnerability to mental states and health issues [13].

Regarding health issues in particular, caregivers may face serious mental health issues, including insomnia, discomfort, despair, stress, and physical problems, as well as emotional disorders such as anxiety and depression [14]. It has been suggested that the caregiver burden experienced by caregivers is associated with increased levels of anxiety, depression, guilt, and distress [15]. In a report involving 1500 American families, the caregivers of dementia patients described experiencing heightened levels of stress, mental and physical health issues, and increased susceptibility to emotional exhaustion [16]. A systematic review revealed that caregivers of dementia patients exhibited a significant prevalence of self-reported depression and compromised physical health, including sleep disturbances, weakened immunity, and an early onset of asthenic syndrome [17].

The primary resource dedicated to patient care is the caregiver’s time. In 2023, approx. 11.5 million individuals caring for people with dementia provided an estimated 18.4 billion hours of unpaid care, averaging about 31 h per caregiver each week, or 1612 h annually. This represents roughly 27 more hours per month compared to caregivers supporting individuals without Alzheimer’s disease or other forms of dementia [18]. Another investigation indicated that caring for a relative with dementia requires an average of 13.8 h per day, compared to just 1.7 h per day for older adults without cognitive impairment [19]. Such substantial time demands were consistent with the greater needs of dementia patients, who experience significantly more difficulties with ADLs and more than twice as many behavioral and memory problems compared to individuals without dementia [20]. It had been reported that caregivers living with an elderly relative in need of care spend an average of 37.4 h per week providing direct assistance, whereas those caring for aging relatives who live separately devote an average of 23.7 h per week to caregiving tasks [16].

Concerning potential economic issues, patients with dementia and their family members often experience financial difficulties related to medical and social expenses, as well as possible loss of income [21].

It was estimated that the global economic costs of dementia in 2019 amounted to USD 1.3 trillion, with approx. 50% of these costs spent on the informal care and supervision that is often provided by relatives and close friends; dementia patients’ caregivers thus frequently experience financial difficulties associated with these costs [2,10]. For example, the value of the informal care for dementia patients in the U.S. totaled $346.585 million in 2023 [18]. According to the WHO, direct medical costs per individual with dementia were estimated at $3864, direct non-medical costs at $8130, and informal care costs at $11,803 [22]. Costs increase as dementia progresses, and the families of patients with severe dementia spend about twice as much annually as those who care for patients with mild dementia [23].

In light of the above-described findings, it is clearly necessary to continue the research concerning dementia patients and their caregivers, and the caregiver burden in particular is an important issue from various perspectives worldwide. Despite a growing body of international literature, the evidence from Central Asia regarding the caregiver burden and psychological distress in dementia remains scarce. There has been an insufficient amount of such research in the Republic of Kazakhstan (hereafter ‘Kazakhstan’), a nation of over 20 million people. Few studies have focused on caregivers in Kazakhstan, and there is also a little consideration of caregivers’ challenges. The several studies of caregivers for the elderly in Kazakhstan focused on elderly individuals in palliative care or those with diabetes, respiratory disease, or stroke [24,25,26,27]. We thus conducted the present study focusing on informal caregivers for dementia patients in Kazakhstan and their caregiver burden, and we investigated the levels of caregiver burden, depression, anxiety, and stress among the caregivers and assessed the relationships between these variables and the caregivers’ socio-demographic characteristics.

We hypothesized that social support for caregivers in Kazakhstan is established based on a clear display of the following items. The research questions concerned (1) the burden and psychological distress of caregivers in Kazakhstan, and (2) the associations between specific indicators of sociodemographic characteristics and more severe levels of caregiver burden, depression, anxiety, and stress.

## 2. Materials and Methods

### 2.1. Study Design and Participants

A quantitative, cross-sectional observational study was conducted using standardized measurement tools to assess caregiver burden and psychological distress. We examined the circumstances of the caregivers of individuals who had received a clinician-confirmed diagnosis of dementia. We defined ‘formal caregivers’ as paid professionals such as nurses, social workers, or home attendants employed to provide care (not immediate-family members or other relatives), and ‘informal caregivers’ as non-paid family members or relatives providing regular care. The study population was informal caregivers. The inclusion criteria for the caregivers were: (*i*) age ≥ 18 years, and (*ii*) being the primary caregiver of a person with a formally established clinical diagnosis of dementia by a licensed psychiatrist or neurologist. The exclusion criteria included (*i*) formal caregiver status, and (*ii*) caregivers of patients with a history of alcoholism, drug addiction, or mental illness other than dementia.

### 2.2. Measurements

We used two validated self-report instruments: the Zarit Burden Interview (ZBI) and the Depression, Anxiety, Stress Scale-21 (DASS-21). The coefficient of internal consistency (Cronbach’s alpha) of the ZBI is 0.93 [28]. The ZBI, which we administered to the caregivers to assess their caregiver burden, is comprised of 22 items that assess the interviewee’s physical, psychological, and social well-being. The answers are given on a 0- to 4-point Likert scale based in the responses ‘never,’ ‘rarely,’ ‘sometimes,’ ‘frequently,’ and ‘nearly always.’ The total possible score thus ranges from 0 to 88 points, with higher scores indicating a higher level of feeling burdened. Total scores of 0–20 points are rated as ‘little-to-no burden;’ 21–40 points as a ‘mild-to-moderate burden,’ 41–60 points as a ‘moderate-to-severe burden’ and 61–88 points as a ‘severe burden.’ We used these descriptions based on previous reports [29,30,31,32].

The DASS-21 questionnaire was used to measure the severity of psychological disorders, depression, anxiety, and stress symptoms among the caregivers. The DASS-21 scale consists of three subscales, each of which is comprised of seven items. The Cronbach’s alpha of the DASS-21 is 0.90. For the three subscales of the DASS-21, the coefficients are ‘depression,’ 0.83; ‘anxiety,’ 0.83; and ‘stress,’ 0.89 [33]. The respondents assessed the intensity of their symptoms over the prior 7 days on a four-point scale ranging from 0 (‘did not apply to me at all’) to 3 points (‘applied to me very much or most of the time’). The minimum and maximum total DASS-21 scores are 0 and 63 points (0–21 points for each subscale). To calculate the final scores, each DASS-21 subscale score is multiplied by 2. These procedures also were based on earlier reports [34,35].

### 2.3. Procedure and Ethical Considerations

This study was conducted between January 2024 and July 2025. The participants were recruited from five facilities: two Regional Mental Health Centers in Kazakhstan’s Abai Region, the East Kazakhstan Regional Mental Health Center, and three Polyclinics in the cities of Semey and Ust-Kamenogorsk. The data were collected in clinical settings (the aforementioned Mental Health Centers and Polyclinics) and the caregivers’ homes. Digital devices were also used to obtain sociodemographic characteristics of some caregivers and to answer any questions or provide assistance. Study participation was voluntary (Table 1), and the candidate participants were informed of the purpose of the study, their right to withdraw at any time, and the confidentiality of their responses. All participants provided written informed consent prior to participation. The study was approved by the Semey Medical University Ethics Committee (Statement no. 2, dated 12 December 2023). The study was adhered to the STROBE guidelines for reporting observational studies [36].

### 2.4. Statistical Analyses

A descriptive analysis was conducted to summarize the sociodemographic and study-specific variables. The distribution of continuous variables (age, ZBI total score, and DASS-21 subscale scores for depression, anxiety, and stress) was tested using the Shapiro–Wilk and Kolmogorov–Smirnov tests, and all of these data significantly deviated from normality (*p* < 0.05). The descriptive results are thus presented as medians (Med.) and interquartile ranges (IQR: Q1–Q3) or ranges, as appropriate. Categorical variables are presented as frequencies and percentages. The sociodemographic variables included sex, ethnic group, marital status, education level, employment status, degree of kinship with the patient, cohabitation, and average time spent caring for the patient per day. The cut-off values for the variables ‘education level’ and ‘average time spent caring for the patient per day’ were pre-specified in order to maximize the clinical interpretability and ensure adequate cell counts.

We collapsed educational categories into ‘higher’ or ‘not higher’ to reflect Kazakhstan’s qualification structure (Bachelor’s/higher or secondary/vocational/college). Preliminary tabulations showed low counts in some granular categories; dichotomization prevented unstable estimates. The >12 h/day threshold operationalizes high-intensity caregiving, indicating the near-continuous supervision and night-time/on-demand assistance that is typical for moderate-to–severe dementia. This binary split also ensured adequate cell counts in our sample. The study-specific variables comprised the severity of caregiver burden based on the ZBI questionnaire and the levels of depression, anxiety, and stress, respectively, based on the DASS-21 scale.

As can be seen in Table 2, each main variable has a subcategory with no signs of caregiver burden or psychological problems, i.e., ‘Little-to-no burden’ for the severity of caregiver burden on the ZBI, and ‘Normal’ for the DASS-21 subscales. For the statistical analysis results presented in Table 3, Table 4, Table 5 and Table 6, we decided to compare two categories of respondents based on the data from Table 2; we divided the respondents into ‘Normal’ (with ‘Little-to-no burden’ for the caregiver burden severity on the ZBI, and ‘Normal’ for the DASS-21 subscales) and ‘Abnormal’ (the remaining subcategories in each of the study-specific variables). We also conducted comparisons within the Abnormal category for each main variable by dividing the caregivers in the Abnormal category into three groups based on the ZBI data (mild-to-moderate, moderate-to-severe, and severe as the caregiver burden) and three groups based on the DASS-21 subscales: mild, moderate, and severe (combining the severe and extremely severe levels) (Table 7, Table 8, Table 9 and Table 10).

Binary logistic regression was used to assess the relationship between the characteristics of the caregivers and the dichotomized outcomes. The outcome was modeled as Normal or Abnormal. For each variable, adjusted odds ratios (AOR) and 95% confidence intervals (CIs) were calculated for the first specified category compared with the reference category, i.e., female sex, ethnic group other than Kazakh, Not married, Not having a higher educational level, No employment, Not a spouse, Not cohabiting with the patient, and >12 h/day spent on caregiving per day. For caregivers in the Abnormal group, ordinal logistic regression with a cumulative logit link (proportional-odds model) was applied, and AORs with 95%CIs were likewise estimated, adjusting for the same covariates (sex, ethnic group, marital status, education level, employment, degree of kinship, cohabitation, and caregiving time per day). The proportional-odds assumption was assessed using the likelihood-ratio (LR) test versus intercept-only; goodness-of-fit (Pearson, Deviance); pseudo-R^2^ (Nagelkerke, McFadden), and the test of parallel lines. Given the potential sparsity of data at the extreme severity levels, multicollinearity was assessed by using variance inflation factor (VIF) and condition indices in an auxiliary linear model for both regressions. All VIFs were <1.6, and condition-index diagnostics showed no concerning clustering, indicating that wide CIs arose from sparse cells rather than collinearity.

The statistical analyses were performed using IBM SPSS Statistics for Windows ver. 27.0 (IBM, Armonk, NY, USA). Missing data were inspected variable-wise with the use of SPSS software (an ‘explore and missing value analysis’). The overall missingness per variable was <1%. A complete-case analysis was also performed. Statistical significance for all of the analyses was set at *p* < 0.05. The minimum required sample size (*n* = 107) was determined a priori with Epi Info software ver. 7.2.7 (U.S. Centers for Disease Control and Prevention, Atlanta, GA, USA). Our achieved sample size (*n* = 174) exceeded this requirement, ensuring sufficient power to detect moderate effect sizes. All statistical analyses were performed using the above-cited SPSS Statistics for Windows ver. 27.0.

## 3. Results

### 3.1. The Sociodemographic Characteristics of the Caregivers

A total of 174 informal caregivers participated in the study. Their characteristics are summarized in Table 1. The median age of the caregivers was 51 years (IQR = 39–63 yrs). The age distribution was significantly different from normal according to both the Shapiro–Wilk and Kolmogorov–Smirnov tests (*p* < 0.05). The majority of the caregivers were female (77.6%) and identified as Kazakh (65.5%). Most of the caregivers were married (64.4%), had a lower level of education rather than higher (66.7%), and were employed (60.3%). The majority of the caregivers (81.6%) were not spouses of the care recipients; spouses thus accounted for 18.4% of the caregivers. Notably, 77.0% of the caregivers cohabited with the care recipients. Regarding the time devoted to caregiving, 78.7% of the caregivers reported spending <12 h/day, whereas about one-fifth (21.3%) spent >12 h/day on caregiving duties.

**Table 1:** Distribution of the caregivers according to their sociodemographic characteristics.

**Variable**		**Med. (Q1–Q3)** **Range**
**Age, yrs**		51 (39–63) 24–78
**Variables**	**Subcategory**	**Subjects,** ***n* (%)**
**Sex**	Male	39 (22.4%)
	Female	135 (77.6%)
**Ethnic group**	Kazakh	114 (65.5%)
	Other ethnic group	60 (34.5%)
**Marital status**	Married	112 (64.4%)
	Not married	62 (35.6%)
**Education level**	Higher	58 (33.3%)
	Not higher	116 (66.7%)
**Employment**	Yes	105 (60.3%)
	No	69 (39.7%)
**Degree of kinship**	Spouse	32 (18.4%)
	Not a spouse	142 (81.6%)
**Cohabitation**	Yes	134 (77.0%)
	No	40 (23.0%)
**Time spent on caregiving per day**	<12 h	137 (78.7%)
	>12 h	37 (21.3%)

### 3.2. The Severity of the Caregiver Burden and the Caregivers’ Psychological States

The severity of caregiver burden and the levels of reported depression, anxiety, and stress among the caregivers are summarized in Table 2. As the distributions of these variables were significantly non-normal (Shapiro–Wilk and Kolmogorov–Smirnov tests, all *p* < 0.05), the results are presented as medians (Med.), interquartile ranges (IQR: Q1–Q3), and ranges. Regarding the severity of caregiver burden as measured by the ZBI, 45.4% of the caregivers described feeling a mild-to-moderate burden, 27.0% reported a moderate-to-severe burden, and 24.1% described little-to-no burden.

The caregivers’ responses on the DASS-21 scale revealed that the depression levels of 64.4% of the caregivers were within the normal range; 16.7% experienced moderate depression symptoms, and 10.9% had mild symptoms. In terms of anxiety, 55.2% of the caregivers were classified as having a normal level of anxiety, with 21.3% experiencing moderate anxiety and 9.2% with mild anxiety. Concerning stress, 71.8% of the caregivers described experiencing stress that was within the normal range, 12.6% exhibited mild stress, and 8.2% experienced severe stress.

The median and IQR values for the ZBI and the DASS-21 subscales depression, anxiety, and stress were 32 (IQR 21–44), 6 (IQR 2–12), 6 (IQR 2–12), and 10 (IQR 6–16) points, respectively.

**Table 2:** Distribution of the severity of caregiver burden and the levels of depression, anxiety, and stress.

Variables	Subcategory	Subjects, *n* (%)	Med. (Q1–Q3) Range
**The severity of caregiver burden (ZBI)**	Little-to-no	42 (24.1%)	32 (21–44) 0–70
	Mild-to-moderate	79 (45.4%)	
	Moderate-to-severe	47 (27.0%)	
	Severe	6 (3.5%)	
**DASS-21 Depression**	Normal	112 (64.4%)	6 (2–12) 0–36
	Mild	19 (10.9%)	
	Moderate	29 (16.7%)	
	Severe	10 (5.7%)	
	Extremely severe	4 (2.3%)	
**DASS-21 Anxiety**	Normal	96 (55.2%)	6 (2–12) 0–28
	Mild	16 (9.2%)	
	Moderate	37 (21.3%)	
	Severe	10 (5.7%)	
	Extremely severe	15 (8.6%)	
**DASS-21 Stress**	Normal	125 (71.8%)	10 (6–16) 0–38
	Mild	22 (12.6%)	
	Moderate	11 (6.3%)	
	Severe	14 (8.2%)	
	Extremely severe	2 (1.2%)	

### 3.3. The Relationships Between the Caregivers’ Sociodemographic Characteristics and the Caregiver Burden and Psychological Distress

Table 3 summarizes the relationships between the caregivers’ sociodemographic characteristics and the severity of the caregiver burden classified herein as Normal and Abnormal. The degree of kinship showed a meaningful association: the caregivers who were spouses of care recipients had significantly higher odds of reporting an abnormal caregiver burden (AOR 2.755, 95%CI: 0.833–9.111), although the confidence interval suggests borderline significance. No significant associations were observed for sex, ethnicity, marital status, education level, employment status, or time spent on caregiving.

**Table 3:** Comparison of the caregiver burden severity categories (Normal, Abnormal) by caregiver characteristics.

Variables	Subcategory	Normal *n* = 42	Abnormal *n* = 132	AOR (95%CI)
**Sex**	Male	11	28	0.857 (0.370–1.985)
	Female	31	104	
**Ethnic group**	Kazakh	31	83	0.674 (0.301–1.510)
	Other ethnic group	11	49	
**Marital status**	Married	30	82	0.622 (0.279–1.387)
	Not married	12	50	
**Educational level**	Higher	14	44	1.038 (0.478–2.251)
	Not higher	28	88	
**Employment**	Yes	27	78	0.991 (0.452–2.173)
	No	15	54	
**Degree of kinship**	Spouse	4	28	2.755 (0.833–9.111)
	Not a spouse	38	104	
**Cohabitation**	Yes	31	103	1.004 (0.411–2.455)
	No	11	29	
**Time spent on caregiving per day**	<12 h	36	101	1.771 (0.641–4.887)
	>12 h	6	31	

Binary logistic regression: The values are **adjusted odds ratios (AORs)** with 95%CIs, adjusted for sex, ethnic group, marital status, education level, employment, degree of kinship, cohabitation, and time spent on caregiving per day. An AOR value >1 indicates greater odds of being in an abnormal category. Model calibration: multicollinearity: all VIF values were <1.3.

Table 4 presents the odds ratios for Normal and Abnormal depression levels across the caregivers’ sociodemographic groups. Most of the variables showed no significant associations with depression levels, but being a spouse of the care recipient was associated with significantly higher odds of depression (AOR 2.825, 95%CI: 1.158–6.890, *p* = 0.022), indicating greater vulnerability among spousal caregivers.

**Table 4:** Comparison of DASS-21 Depression levels in the Normal and Abnormal caregiver categories by caregiver characteristics.

Variables	Subcategory	Normal *n* = 112	Abnormal *n* = 62	AOR (95%CI)
**Sex**	Male	29	10	0.522 (0.227–1.203)
	Female	83	52	
**Ethnic group**	Kazakh	75	39	0.897 (0.448–1.792)
	Other ethnic group	37	23	
**Marital status**	Married	72	40	0.834 (0.406–1.715)
	Not married	40	22	
**Educational level**	Higher	36	22	1.128 (0.562-2.262)
	Not higher	76	40	
**Employment**	Yes	68	37	1.332 (0.652–2.719)
	No	44	25	
**Degree of kinship**	Spouse	14	18	2.825 (1.158–6.890) *
	Not a spouse	98	44	
**Cohabitation**	Yes	82	52	1.974 (0.820–4.751)
	No	30	10	
**Time spent on caregiving per day**	<12 h	87	50	0.633 (0.275–1.461)
	>12 h	25	12	

Binary logistic regression: The values are **adjusted odds ratios (AORs)** with 95%CIs, adjusted for sex, ethnic group, marital status, education level, employment, degree of kinship, cohabitation, and time spent on caregiving per day. An AOR value > 1 indicates greater odds of being in an abnormal category. Model calibration: multicollinearity: all VIF values were <1.3. * *p* < 0.05.

The degree of kinship again emerged as a significant predictor across anxiety levels. Spousal caregivers had significantly higher odds of experiencing anxiety (AOR 2.925, 95%CI: 1.188–7.202, *p* = 0.020). The caregivers who did not report having a higher education level had a significantly greater risk of being in the Abnormal category (AOR 0.470, 95%CI: 0.236–0.938, *p* = 0.029) as shown in Table 5.

**Table 5:** Comparison of DASS-21 Anxiety levels in the Normal and Abnormal caregiver categories by caregiver characteristics.

Variables	Subcategory	Normal *n* = 96	Abnormal *n* = 78	AOR (95%CI)
**Sex**	Male	22	17	0.942 (0.444–1.988)
	Female	74	61	
**Ethnic group**	Kazakh	63	51	1.160 (0.595–2.261)
	Other ethnic group	33	27	
**Marital status**	Married	63	49	0.628 (0.315–1.254)
	Not married	33	29	
**Educational level**	Higher	38	20	0.470 (0.236–0.938) *
	Not higher	58	58	
**Employment**	Yes	59	46	1.298 (0.657–2.565)
	No	37	32	
**Degree of kinship**	Spouse	12	20	2.925 (1.188–7.202) *
	Not a spouse	84	58	
**Cohabitation**	Yes	72	62	1.202 (0.533–2.709)
	No	24	16	
**Time spent on caregiving per day**	<12 h	74	63	0.651 (0.291–1.454)
	>12 h	22	15	

Binary logistic regression: The values are **adjusted odds ratios (AORs)** with 95%CIs, adjusted for sex, ethnic group, marital status, education level, employment, degree of kinship, cohabitation, and time spent on caregiving per day. An AOR value > 1 indicates greater odds of being in abnormal category. Model calibration: multicollinearity: all VIF values were <1.3. * *p* < 0.05.

Regarding stress levels, the analyses revealed that the caregivers who were spouses of care recipients (AOR 2.082, 95%CI: 0.850–5.100) had higher odds of stress in the Abnormal category, although this result was not significant. No significant associations were observed for sex, ethnicity, marital status, education level, employment status, or time spent on caregiving. These findings are shown in Table 6.

**Table 6:** Comparison of DASS-21 Stress levels in the Normal and Abnormal caregiver categories by caregiver characteristics.

Variables	Subcategory	Normal *n* = 125	Abnormal *n* = 49	AOR (95%CI)
**Sex**	Male	28	11	1.006 (0.434–2.282)
	Female	97	38	
**Ethnic group**	Kazakh	83	31	0.895 (0.435–1.842)
	Other ethnic group	42	18	
**Marital status**	Married	78	34	1.190 (0.554–2.556)
	Not married	47	15	
**Educational level**	Higher	42	16	1.046 (0.502–2.180)
	Not higher	83	33	
**Employment**	Yes	78	27	0.905 (0.436–1.876)
	No	47	22	
**Degree of kinship**	Spouse	18	14	2.082 (0.850–5.100)
	Not a spouse	107	35	
**Cohabitation**	Yes	94	40	1.152 (0.458–2.896)
	No	31	9	
**Time spent on caregiving per day**	<12 h	99	38	1.036 (0.441–2.434)
	>12 h	26	11	

Binary logistic regression: The values are **adjusted odds ratios (AORs)** with 95%CIs, adjusted for sex, ethnic group, marital status, education level, employment, degree of kinship, cohabitation, and time spent on caregiving per day. An AOR value > 1 indicates greater odds of being in an abnormal category. Model calibration: multicollinearity: all VIF values were <1.3.

The results of the multilevel comparison of caregiver burden across three categories (mild-to-moderate, moderate-to-severe, and severe burden) revealed that the degree of kinship was strongly associated with a higher caregiver burden. Spouses had significantly increased odds of a severe burden (AOR 2.667, 95%CI: 1.038–6.855, *p* = 0.042). These data are presented in Table 7.

**Table 7:** Comparison of abnormal ZBI levels by caregiver characteristics.

Variables	Subcategory	Mild-to- Moderate *n* = 79	Moderate-to- Severe *n* = 47	Severe *n* = 6	AOR (95%CI)
**Sex**	Male	17	10	1	0.906 (0.371–2.210)
	Female	62	37	5	
**Ethnic group**	Kazakh	48	30	5	1.385 (0.641–2.995)
	Other ethnic group	31	17	1	
**Marital status**	Married	47	31	4	0.998 (0.449–2.217)
	Not married	32	16	2	
**Educational level**	Higher	27	15	2	0.916 (0.414–2.024)
	Not higher	52	32	4	
**Employment**	Yes	49	27	2	0.976 (0.450–2.117)
	No	30	20	4	
**Degree of kinship**	Spouse	11	14	3	2.667 (1.038–6.855) *
	Not a spouse	68	33	3	
**Cohabitation**	Yes	57	40	6	2.088 (0.738–5.906)
	No	22	7	0	
**Time spent on caregiving per day**	<12 h	60	38	3	1.305 (0.541–3.152)
	>12 h	19	9	3	

Proportional-odds ordinal logistic regression (logit link). The values are adjusted odds ratios (AORs) with 95%CIs, adjusted for sex, ethnic group, marital status, education level, employment, degree of kinship, cohabitation, and time spent on caregiving per day. An AOR value > 1 indicates greater odds of being in a higher severity category. Model fit: likelihood-ratio χ^2^ = 10.328, df = 8, *p* = 0.243; Pearson χ^2^ *p* = 0.691; Deviance χ^2^ *p* = 0.732; pseudo-R^2^ (Nagelkerke) = 0.094; McFadden = 0.048. The proportional-odds assumption not violated (test of parallel lines χ^2^ = 6.466, df = 8, *p* = 0.595). Multicollinearity was checked in an auxiliary linear model (all VIF values were <1.3). * *p* < 0.05.

The results in Table 8 demonstrated that the caregivers’ educational attainment correlated with the severity of depression. Caregivers without higher educational levels were significantly more likely to report severe depression symptoms compared to those with higher education (AOR 0.243, 95%CI: 0.075–0.785, *p* = 0.018).

**Table 8:** Comparison of DASS-21 abnormal Depression levels by caregiver characteristics.

Variables	Subcategory	Mild *n* = 19	Moderate *n* = 29	Severe *n* = 14	AOR (95%CI)
**Sex**	Male	1	7	2	1.455 (0.393–5.382)
	Female	18	22	12	
**Ethnic group**	Kazakh	13	18	8	0.613 (0.212–1.772)
	Other ethnic group	6	11	6	
**Marital status**	Married	12	20	8	0.656 (0.214–2.016)
	Not married	7	9	6	
**Educational level**	Higher	11	8	3	0.243 (0.075–0.785) *
	Not higher	8	21	11	
**Employment**	Yes	13	17	7	0.707 (0.242–2.061)
	No	6	12	7	
**Degree of kinship**	Spouse	5	8	5	0.800 (0.229–2.795)
	Not a spouse	14	21	9	
**Cohabitation**	Yes	16	23	13	1.804 (0.406–8.012)
	No	3	6	1	
**Time spent on caregiving per day**	<12 h	16	22	12	1.548 (0.415–5.772)
	>12 h	3	7	2	

Proportional-odds ordinal logistic regression (logit link). The values are adjusted odds ratios (AORs) with 95%CIs, adjusted for sex, ethnic group, marital status, education level, employment, degree of kinship, cohabitation, and time spent on caregiving per day. An AOR value > 1 indicates greater odds of being in a higher severity category. Model fit: likelihood-ratio χ^2^ = 8.794, df = 8, *p* = 0.360; Pearson χ^2^ *p* = 0.013; Deviance χ^2^ *p* = 0.041; pseudo-R^2^ (Nagelkerke) = 0.151; McFadden = 0.067. The proportional-odds assumption was not violated (test of parallel lines χ^2^ = 6.046, df = 8, *p* = 0.642). Multicollinearity was checked in an auxiliary linear model (all VIF values were <1.5). * *p* < 0.05.

As presented in Table 9, a strong association was again noted between spousal kinship and elevated anxiety levels. The odds of experiencing more severe anxiety symptoms were over five times higher among the spouse caregivers (AOR 5.795, 95%CI: 1.649–20.348, *p* = 0.006). The caregivers who were employed also showed a significantly higher risk of having a more severe anxiety level (AOR 2.765, 95%CI: 1.030–7.426, *p* = 0.044).

**Table 9:** Comparison of DASS-21 abnormal Anxiety levels by caregiver characteristics.

Variables	Subcategory	Mild *n* = 16	Moderate *n* = 37	Severe *n* = 25	AOR (95%CI)
**Sex**	Male	3	7	7	1.952 (0.671–5.675)
	Female	13	30	18	
**Ethnic group**	Kazakh	12	24	15	0.566 (0.206–1.553)
	Other ethnic group	4	13	10	
**Marital status**	Married	12	20	17	0.716 (0.273–1.881)
	Not married	4	17	8	
**Educational level**	Higher	3	10	7	1.824 (0.628–5.307)
	Not higher	13	27	18	
**Employment**	Yes	6	24	16	2.765 (1.030–7.426) *
	No	10	13	9	
**Degree of kinship**	Spouse	3	6	11	5.795 (1.649–20.348) **
	Not a spouse	13	31	14	
**Cohabitation**	Yes	12	28	22	1.078 (0.321–3.622)
	No	4	9	3	
**Time spent on caregiving per day**	<12 h	15	29	19	0.348 (0.104–1.1721)
	>12 h	1	8	6	

Proportional-odds ordinal logistic regression (logit link). The values are adjusted odds ratios (AORs) with 95%CIs, adjusted for sex, ethnic group, marital status, education level, employment, degree of kinship, cohabitation, and time spent on caregiving per day. An AOR value > 1 indicates greater odds of being in a higher severity category. Model fit: likelihood-ratio χ^2^ = 15.993, df = 8, *p* = 0.042; Pearson χ^2^ *p* = 0.266; Deviance χ^2^ *p* = 0.319; pseudo-R^2^ (Nagelkerke) = 0.212; McFadden = 0.098. The proportional-odds assumption was not violated (test of parallel lines χ^2^ = 6.637, df = 8, *p* = 0.576). Multicollinearity was checked in an auxiliary linear model (all VIF values were <1.5). * *p* < 0.05, ** *p* < 0.01.

The associations between stress severity and sociodemographic characteristics are described by the data in Table 10. Employment was revealed as a significant protective factor, with unemployed caregivers significantly more likely to experience higher stress levels (AOR 0.154, 95%CI: 0.033–0.731, *p* = 0.019). In addition, being a spouse of the care recipient (AOR 3.274, 95%CI: 0.672–15.297) and cohabiting with the recipient (AOR 1.395, 95%CI: 0.190–10.247) were associated with increased stress levels, although not significantly.

**Table 10:** Comparison of DASS-21 abnormal Stress levels by caregiver characteristics.

Variables	Subcategory	Mild *n* = 22	Moderate *n* = 11	Severe *n* = 16	AOR (95%CI)
**Sex**	Male	6	4	1	0.679 (0.152–3.043)
	Female	16	7	15	
**Ethnic group**	Kazakh	13	7	11	2.686 (0.628–11.485)
	Other ethnic group	9	4	5	
**Marital status**	Married	14	10	10	0.262 (0.052–1.320)
	Not married	8	1	6	
**Educational level**	Higher	9	5	2	0.281 (0.067–1.183)
	Not higher	13	6	14	
**Employment**	Yes	16	5	6	0.154 (0.033–0.731) *
	No	6	6	10	
**Degree of kinship**	Spouse	3	4	7	3.274 (0.672–15.927)
	Not a spouse	19	7	9	
**Cohabitation**	Yes	15	10	15	1.395 (0.190–10.247)
	No	7	1	1	
**Time spent on caregiving per day**	<12 h	19	8	11	0.223 (0.039–1.260)
	>12 h	3	3	5	

Proportional-odds ordinal logistic regression (logit link). The values are adjusted odds ratios (AORs) with 95%CIs, adjusted for sex, ethnic group, marital status, education level, employment, degree of kinship, cohabitation, and time spent on caregiving per day. An AOR value > 1 indicates greater odds of being in a higher severity category. Model fit: likelihood-ratio χ^2^ = 19.378, df = 8, *p* = 0.013; Pearson χ^2^ *p* = 0.413; Deviance χ^2^ *p* = 0.466; pseudo-R^2^ (Nagelkerke) = 0.371; McFadden = 0.186. The proportional-odds assumption was not violated (test of parallel lines χ^2^ = 8.100, df = 8, *p* = 0.424). Multicollinearity was checked in an auxiliary linear model (all VIF values were <1.6). * *p* < 0.05.

## 4. Discussion

We assessed the severity of caregiver burden and psychological distress among informal caregivers of individuals with dementia in Kazakhstan. A considerable proportion of the caregivers reported a burden and symptoms of depression, anxiety, and stress, and their data indicate that dementia caregiving imposes a substantial emotional and physical toll on informal caregivers. Our findings are consistent with those of international studies [14,21,37] and contribute valuable region-specific insights to the global understanding of dementia caregiving, particularly in Central Asia, where the number of such studies is limited. In this study, approx. 76% of the caregivers reported experiencing some level of burden, which aligns with global trends showing a high prevalence of burden among dementia caregivers [38,39,40]. These results are consistent with research that has consistently emphasized that caregiving correlates with an increased risk of mental fatigue [39,41,42,43]. Psychological support and relief for these caregivers are thus very important.

Most of the caregivers in our study were married and more than half were employed, reflecting the international evidence that caregiving is often undertaken by individuals who simultaneously balance family responsibilities and professional work obligations [44,45]. The vast majority of the present study’s caregivers cohabited with the care recipient, aligning with the international finding that co-residing is a common normative arrangement facilitating sustained caregiving [20,46]. Caregivers need to take time off to relax physically and mentally, and they can feel reassured if other family members, relatives, or social services are available to provide care during periods when the main caregiver is away.

The pattern that emerged most consistently across the binary and ordinal models in our present analyses is the elevated vulnerability of spousal caregivers. The spouses had lower odds of being in the normal range on the depression and anxiety DASS-21 domains and higher cumulative odds of more severe abnormal states in the within-abnormal analysis of anxiety. This is concordant with recent review data indicating that spousal caregivers were at heightened risk for depression relative to other kin of dementia patients, possibly due to the cumulative emotional, relational, and instrumental demands that are inherent to caring for a life partner (e.g., intimacy care, decision burden, and role loss) [47,48].

Our analyses also identified two factors that had a protective effect against the caregiver burden. First, higher education was associated with lower odds of being in an abnormal anxiety category, and having more severe depression was classified as the abnormal state, which is consistent with observations that greater educational attainment may confer coping resources (e.g., health literacy, problem-solving, navigation skills) that buffer affective symptoms [49]. Second, employment showed a protective association with stress severity (ordinal model), thus aligning with emerging studies that link employment to better caregiver mental health, possibly through social contact, identity continuity, and structured respite from caregiving, provided that the caregiver’s work–care balance is supported [50]. The design of caregiver support programs requires viewpoints of not only psychiatric aspects but also social support.

Our data also revealed a reverse situation regarding anxiety, in which the caregivers who were employed had a higher risk of having more severe levels of anxiety, which may be due to the difficulty in finding the right balance between work and caring responsibilities [51]. In this regard, it is important to have a vacation system that allows employed caregivers to take leave from their jobs with peace of mind and for the workplace to understand the caregiver’s challenges.

Although the time spent on caregiving per day did not show a significant association with the caregiver burden or psychological outcomes, over 20% of the caregivers in this study reported caregiving durations exceeding 12 h/day. This is consistent with prior reports indicating that dementia caregivers spend more time on caregiving compared to those assisting patients without cognitive impairments [3,20]. These findings also highlight the necessity of providing rest periods and social services to caregivers, in light of their physical and mental exhaustion.

Our search of the relevant literature indicates that this study is among the first to explore the caregiver burden of individuals who provide care for patients with dementia in Kazakhstan by using standardized psychological measures. Our findings demonstrate that the challenges faced by dementia caregivers in Kazakhstan are consistent with global patterns but may be exacerbated by local systemic limitations. Kazakhstan, like many countries in Central Asia, lacks formalized structures for dementia-specific long-term care, and informal caregiving remains the mainstay of support.

Our findings and those of other investigations indicate that the observed burden among Kazakh caregivers reflects the intersection of cultural expectations, a lack of respite services, and limited public funding. In many post-Soviet societies such as Kazakhstan, caring for elderly parents or spouses is seen as a moral and familial obligation; this can hinder caregivers’ readiness to seek outside help, even when the caregiver strain becomes unsustainable. The high prevalence of caregiver burden and psychological symptoms revealed by our study emphasizes the need for routine psychological assessments and support for dementia caregivers in Kazakhstan. Despite the growing recognition of caregiving challenges globally, support services in many low- and middle-income countries, including Kazakhstan, remain fragmented and insufficient. In addition, caregivers, especially spouses, should be prioritized for targeted interventions, including psychoeducation, support groups, and respite services. The integration of these interventions into national healthcare policy is essential. The fields of study and organizations that consider the burden of caregiving for dementia patients must therefore act together to tackle this issue.

Our study has both strengths and limitations. It is one of the first to explore the caregiver burden and psychological distress among dementia caregivers in Kazakhstan by using validated screening instruments. However, the study’s cross-sectional design limits causal inferences. Longitudinal studies are necessary to clarify how the caregiver burden evolves over time, especially with the progression of dementia. Second, self-reported data (as provided by our study sample) are subject to social desirability and recall biases, particularly in societies in which mental health issues are stigmatized. Third, although our sample was recruited from multiple clinical and community sources, it may not be representative of all caregivers in Kazakhstan, particularly those in rural or underserved regions. Fourth, we did not capture relationship details beyond the spouse/non-spouse dichotomy (e.g., child, daughter-in-law, sibling) or patient severity indicators (e.g., global cognition, ADLs, and IADLs) or behavioral and psychological symptoms of dementia (BPSD). Future research should incorporate these variables to provide a more nuanced understanding of caregiver experiences.

Prospective longitudinal studies are desired to provide richer insights into the progression of the caregiver burden. We believe that further detailed research is also important for understanding caregivers’ coping mechanisms and support needs. Finally, qualitative studies exploring cultural perceptions of caregiving in Kazakhstan could inform the development of more culturally responsive interventions. Our present findings are useful for the great number of other countries that need to conduct similar research.

## 5. Conclusions

The results of this study demonstrate that psychological distress and caregiver burden are widespread among informal dementia caregivers in Kazakhstan, with spousal caregiving emerging as the most consistent correlate of worse outcomes. Given the limited availability of formal dementia care in Kazakhstan, it is essential to strengthen the country’s informal caregiver support systems. Prioritizing high-burden groups—particularly spouses—within national dementia-care strategies can help safeguard caregivers’ well-being and improve the quality of care provided to the growing number of individuals with dementia.

## Data Availability

Data are contained within the article.

## References

[1] Statistics Bureau, Ministry of Internal Affairs and Communications (2025). World Statistics 2025.

[2] World Health Organization (2025). Dementia.

[3] Freedman V.A., Patterson S.E., Cornman J.C., Wolff J.L. (2022). A day in the life of caregivers to older adults with and without dementia: Comparisons of care time and emotional health. Alzheimer’s Dement..

[4] Alzheimer’s Association Report (2023). 2023 Alzheimer’s disease facts and figures. Alzheimer’s Dement..

[5] Zarit S.H., Todd P.A., Zarit J.M. (1986). Subjective burden of husbands and wives as caregivers: A longitudinal study. Gerontologist.

[6] Cartaxo A., Koller M., Mayer H., Kolland F., Nagl-Cupal M. (2023). Risk Factors with the Greatest Impact on Caregiver Burden in Informal Homecare Settings in Austria: A Quantitative Secondary Data Analysis. Health Soc. Care Community.

[7] Soh X.C., Hartanto A., Ling N., Reyes M., Sim L., Majeed N.M. (2025). Prevalence of depression, anxiety, burden, burnout, and stress in informal caregivers: An umbrella review of meta-analyses. Arch. Gerontol. Geriatr. Plus.

[8] Loo Y.X., Yan S., Low L.L. (2022). Caregiver burden and its prevalence, measurement scales, predictive factors and impact: A review with an Asian perspective. Singapore Med. J..

[9] Nasreen H.E., Tyrrell M., Vikström S., Craftman Å., Syed Ahmad S.A.B., Zin N.M., Aziz K.H.A., Mohd Tohit N.B., Md Aris M.A., Kabir Z.N. (2024). Caregiver burden, mental health, quality of life and self-efficacy of family caregivers of persons with dementia in Malaysia: Baseline results of a psychoeducational intervention study. BMC Geriatr..

[10] Kim B., Noh G.O., Kim K. (2021). Behavioural and psychological symptoms of dementia in patients with Alzheimer’s disease and family caregiver burden: A path analysis. BMC Geriatr..

[11] Nakabe T., Sasaki N., Uematsu H., Kunisawa S., Wimo A., Imanaka Y. (2019). Classification tree model of the personal economic burden of dementia care by related factors of both people with dementia and caregivers in Japan: A cross-sectional online survey. BMJ Open.

[12] Chekani F., Pike J., Jones E., Husbands J., Khandker R.K. (2021). Impact of Dementia-Related Behavioral Symptoms on Healthcare Resource Use and Caregiver Burden: Real-World Data from Europe and the United States. J. Alzheimer’s Dis..

[13] Monteiro B.C.D.C., Dos Santos T.T.B.A., Nogueira M.M.L., Dourado M.C.N. (2023). The relationship between burden and caregiver’s sleep disturbances in dementia: A systematic review. Dement. Neuropsychol..

[14] Hu H., Hu X., Xu Y. (2023). Caring load and family caregivers’ burden in China: The mediating effects of social support and social exclusion. Front. Public Health.

[15] Del-Pino-Casado R., Priego-Cubero E., López-Martínez C., Orgeta V. (2021). Subjective caregiver burden and anxiety in informal caregivers: A systematic review and meta-analysis. PLoS ONE.

[16] Samuels C. Caregiver Statistics: A Data Portrait of Family Caregiving. A Place for Mom: New York, NY, USA. https://www.aplaceformom.com/senior-living-data/articles/caregiver-statistics.

[17] Xiong C., Biscardi M., Astell A., Nalder E., Cameron J.I., Mihailidis A., Colantonio A. (2020). Sex and gender differences in caregiving burden experienced by family caregivers of persons with dementia: A systematic review. PLoS ONE.

[18] Alzheimer’s Association Report (2024). 2024 Alzheimer’s disease facts and figures. Alzheimer’s Dement..

[19] Galske J., Chera T., Hwang U., Monin J.K., Venkatesh A., Lam K., Leggett A.N., Gettel C. (2024). Daily care hours among caregivers of older emergency department patients with dementia and undiagnosed cognitive impairment. J. Am. Geriatr. Soc..

[20] Sheehan O.C., Haley W.E., Howard V.J., Huang J., Rhodes J.D., Roth D.L. (2021). Stress, Burden, and Well-being in Dementia and Nondementia Caregivers: Insights from the Caregiving Transitions Study. Gerontologist.

[21] Hellis E., Mukaetova-Ladinska E.B. (2022). Informal Caregiving and Alzheimer’s Disease: The Psychological Effect. Medicina.

[22] World Health Organization (2021). Global Status Report on the Public Health Response to Dementia.

[23] Yan X., Li F., Chen S., Jia J. (2019). Associated Factors of Total Costs of Alzheimer’s Disease: A Cluster-Randomized Observational Study in China. J. Alzheimer’s Dis..

[24] Zhumagaliyeva A., Ottaviani S., Greulich T., Gorrini M., Vogelmeier C., Karazhanova L., Nurgazina G., DeSilvestri A., Kotke V., Barzon V. (2017). Case-finding for alpha1-antitrypsin deficiency in Kazakh patients with COPD. Multidiscip. Respir. Med..

[25] Salikhanov I., Katapodi M.C., Kunirova G., Crape B.L. (2023). Improving palliative care outcomes in remote and rural areas of LMICs through family caregivers: Lessons from Kazakhstan. Front. Public Health.

[26] Miranda J.C., Raza S.A., Kolawole B., Khan J.K., Alvi A., Ali F.S., Chukwudi E.E., Ram N., Oluwatoyin A. (2023). Enhancing Diabetes Care in LMICs: Insights from a Multinational consensus. Pak. J. Med. Sci..

[27] Kairatova G.K., Khismetova Z.A., Smailova D.S., Serikova-Esengeldina D.S., Berikuly D., Akhmetova K.M., Shalgumbayeva G.M. (2024). Assessment of Skillsof Caregivers Providing Care for Stroke Patients in East Kazakhstan Region. Healthcare.

[28] Lutova N.B., Makarevich O.V., Sorokin M.Y., Khobeysh M.A. (2023). Validation of the Zarit Burden Interview (Zbi) in Caregivers of Patients with Schizophrenia Spectrum Disorder in Russian-Speaking Sample. J. Soc. Clin. Psychiatry.

[29] A Courtesy of L’Orech Yomim/Center for Healthy Living, Inc Zarit Burden Interview Assessing Caregiver Burden. https://wai.wisc.edu/wp-content/uploads/sites/1129/2021/11/Zarit-Caregiver-Burden-Assessment-Instruments.pdf.

[30] Kršíková T., Zeleníková R. (2018). Association between burden and depression in caregivers of dementia patients. Cent. Eur. J. Nurs. Midw..

[31] Ledda C., Montanaro E., Imbalzano G., Merola A., Bruno I., Artusi C.A., Zibetti M., Rizzone M.G., Bozzali M., Sobrero G. (2022). Burden of caregiving for cardiovascular dysautonomia in Parkinson’s disease. Clin. Auton. Res..

[32] Rego Filho J.F., Sena C., Wajnsztejn R. (2023). Burden in caregivers of children with congenital Zika syndrome in Pernambuco, Brazil: Analysis and application of the Zarit burden interview scale. PeerJ.

[33] Ruzhenkova V.V., Ruzhenkov V.A. (2019). Russian Adaptation of the Dass-21 for Screening and Diagnosis of Depression, Anxiety and Stress. Bull. Neurol. Psychiatry Neurosurg..

[34] Ramón-Arbués E., Gea-Caballero V., Granada-López J.M., Juárez-Vela R., Pellicer-García B., Antón-Solanas I. (2020). The Prevalence of Depression, Anxiety and Stress and Their Associated Factors in College Students. Int. J. Environ. Res. Public Health.

[35] de Araujo E.L., Rodrigues M.R., Kozasa E.H., Lacerda S.S. (2023). Psychoeducation versus psychoeducation integrated with yoga for family caregivers of people with Alzheimer’s disease: A randomized clinical trial. Eur. J. Ageing.

[36] STROBE STROBE Checklists. https://www.strobe-statement.org/checklists/.

[37] Wang L., Zhou Y., Fang X., Qu G. (2022). Care burden on family caregivers of patients with dementia and affecting factors in China: A systematic review. Front. Psychiatry.

[38] Manee F., Alnaser M.Z., Alqattan A., Almutairi S., Maqtouf H. (2025). A Comparative Study of Burden of Care, Anxiety, and Well-Being Among Family Caregivers of Elderly with Dementia: Evidence from Kuwait. Healthcare.

[39] Abdelhalim D.S., Ahmed M.M., Hussein H.A., Khalaf O.O., Sarhan M.D. (2024). Burden of Care, Depression, and Anxiety Among Family Caregivers of People with Dementia. J. Prim. Care Community Health.

[40] Shim Y.S., Park K.H., Chen C., Dominguez J.C., Kang K., Kim H.J., Hong Z., Lin Y.T., Chu L.W., Jung S. (2021). Caregiving, care burden and awareness of caregivers and patients with dementia in Asian locations: A secondary analysis. BMC Geriatr..

[41] Lee Y., Xu L., Kim B.J., Chen L. (2020). Leisure activity, gender and depressive symptoms among dementia caregivers: Findings from the REACH II. Aging Ment. Health.

[42] Smyth A., Whitehead L., Quigley E., Vafeas C., Emery L. (2020). Disrupted sleep and associated factors in Australian dementia caregivers: A cross-sectional study. BMC Geriatr..

[43] Zahed S., Emami M., Eslami A.A., Barekatain M., Hassanzadeh A., Zamani-Alavijeh F. (2020). Stress as a challenge in promoting mental health among dementia caregivers. J. Educ. Health Promot..

[44] Huang H.L., Liao Y.T., Kung P.C., Shyu Y.L., Hsu W.C., Hsu J.L. (2024). Caregiving management needs and predictors for family caregivers of persons with dementia: A cross-sectional study. BMC Geriatr..

[45] Wolff J.L., Cornman J.C., Freedman V.A. (2025). The Number of Family Caregivers Helping Older US Adults Increased From 18 Million to 24 Million, 2011–2022. Health Aff..

[46] Froelich L., Lladó A., Khandker R.K., Pedrós M., Black C.M., Sánchez Díaz E.J., Chekani F., Ambegaonkar B. (2021). Quality of Life and Caregiver Burden of Alzheimer’s Disease Among Community Dwelling Patients in Europe: Variation by Disease Severity and Progression. J. Alzheimer’s Dis. Rep..

[47] Chen C., Thunell J., Zissimopoulos J. (2020). Changes in physical and mental health of Black, Hispanic, and White caregivers and non-caregivers associated with onset of spousal dementia. Alzheimer’s Dement..

[48] Sallim A.B., Sayampanathan A.A., Cuttilan A., Ho R. (2015). Prevalence of Mental Health Disorders Among Caregivers of Patients with Alzheimer Disease. J. Am. Med. Dir. Assoc..

[49] Carpinelli Mazzi M., Iavarone A., Musella C., De Luca M., de Vita D., Branciforte S., Coppola A., Scarpa R., Raimondo S., Sorrentino S. (2020). Time of isolation, education and gender influence the psychological outcome during COVID-19 lockdown in caregivers of patients with dementia. Eur. Geriatr. Med..

[50] Scheuermann J.S., Pendergrass A., Diehl K., Herr R.M. (2024). The roles of employment status and income in the mental health of informal caregivers in Germany. BMC Public Health.

[51] Hastert T.A., Ruterbusch J.J., Nair M., Noor M.I., Beebe-Dimmer J.L., Schwartz K., Baird T.E., Harper F.W.K., Thompson H., Schwartz A.G. (2020). Employment Outcomes, Financial Burden, Anxiety, and Depression Among Caregivers of African American Cancer Survivors. JCO Oncol. Pract..

